# Neurobehavioural and cognitive effects of prenatal exposure to organochlorine compounds in three year old children

**DOI:** 10.1186/s12887-021-02533-2

**Published:** 2021-02-26

**Authors:** Griet Vermeir, Adrian Covaci, Nik Van Larebeke, Greet Schoeters, Vera Nelen, Gudrun Koppen, Mineke Viaene

**Affiliations:** 1 Neurotoxicology Expertise Center, Public psychiatric care centre Geel, Pas 200, 2440 Geel, Belgium; 2 Toxicological Centre, University of Antwerp, Campus Drie Eiken ,DS551, Universiteitsplein 1, 2610 Wilrijk, Belgium; 3 Department of Radiotherapy and Experimental Cancerology, Ghent University, De Pintelaan 185, 9000 Gent, Belgium; 4 Analytical, Environmental and Geochemistry, Vrije Universiteit Brussel, Pleinlaan 2, 1050 Brussels, Belgium; 5 Vlaamse Instelling voor Technologisch Onderzoek (VITO), Environmental Toxicology Unit, Mol, Belgium; 6 Provinciaal Institut voor Hygiëne, Kronenburgstraat 45, 3000 Antwerpen, Belgium; 7 Departement of Environment and Health, faculty of medicine, KU Leuven, Kapucijnenvoer 35-D-box 7001, 3000 Leuven, Belgium; 8 Department of Neurology, General Hospital Geel, J.B. Steffensstraat 2, 2440 Geel, Belgium

**Keywords:** Polychlorinated biphenyls (PCBs), P,p’-dichlorodiphenyldichloroethylene (DDE), Hexachlorobenzene (HCB), Dioxin-like substances, Cord blood, Mental development, Motor development, Play behaviour, Neutral (non-gender) behaviour

## Abstract

**Background:**

We report data of a Belgian observational prospective cohort study regarding cognitive and behavioural development until the age of 36 months in relation to internal exposure to organochlorine pollutants [sum of polychlorinated biphenyls (sum PCB), dioxin-like activity, PCB118, PCB170, hexachlorobenzene (HCB) and p,p’-dichlorodiphenyldichloroethylene (DDE)] measured in cord blood.

**Methods:**

Participants were recruited as part of an Flemish Environmental Health Survey (2002–2006). Two hundred and six mother-child pairs were recruited. Hundred twenty five toddlers [Reynell Taal Ontwikkelings Schalen (language development, RTOS), Snijders-Oomen Niet-verbale intelligentietest (non-verbal intelligence, SON), Bayley Scales, milestones, Infant Behaviour Questionnaire (IBQ), gender specific play behaviour, Neurobehavioral Evaluation System (NES)-attentional task] and their mothers [Home Observation Measurement of the Environment (HOME), Wechsler Abbreviated Scale of Intelligence (WASI), State-Trait Anxiety Inventory (STAI), general questionnaires] were tested. Statistical analysis was performed with the SPSS program. Much attention was paid to confounding factors.

**Results:**

In the first years of development, higher organochlorine pollutants were associated with less active children (delayed crawling: sum PCB*HCB (*p* < 0.05), sumPCB*DDE (*p* < 0.1); delayed first steps alone: sum PCB (*p* < 0.5), PCB118 (*p* < 0.01), PCB170 (p < 0.01), HCB (p < 0.01); less switching between toys: sum PCB (p < 0.01); less switching between toys in boys: PCB118 (p < 0.01), sum PCB(p < 0.01)). At 12 months children with higher dioxin-like activity tended to show less fear responses(*p* < 0.1) (IBQ 12 months). At 36 months, a slower development of language comprehension (RTOS) was related to all organochlorine exposure parameters(p < 0.1 or *p* < 0.05) except DDE. Lower nonverbal IQ scores (SON) were related to PCB118 in boys only(p < 0.05 or p < 0.01). Less masculine and more non-gender specific play behaviour was associated with sum PCB in boys and girls at 36 months(p < 0.1). Moreover, PCB118 (p < 0.05), PCB170 (p < 0.1), HCB(p < 0.05) and DDE(p < 0.05) were associated with diminished masculine play behaviour in boys.

**Conclusion:**

Our data confirm the observations that neurobehavioral development of young children is adversely influenced by environmental concentrations of PCBs, especially in boys. In this context, observation of play behaviour seems to be a reliable, easy to perform and sensitive test to detect neurotoxic effects of chemicals like PCB’s and dioxin-like compounds in very young children. On the basis of our results, we hypothesize that an underarrousal pattern may play a role in the spectrum of effects measured in toddlers prenatally exposed to PCBs and dioxin-like compounds.

**Supplementary Information:**

The online version contains supplementary material available at 10.1186/s12887-021-02533-2.

## Background

PCBs (polychlorinated biphenyls) are classified as POPs (persistent organic pollutants) due to their very stable chemical structures and consequently very long half lives in the environment and living species, including humans [1]. Therefore the handling of PCBs has been strictly regulated in many countries for quite some time. Nevertheless, they will persist in many surroundings for some more decades and will stay a potential health hazard. Human exposure to PCBs is largely due to ingestion of contaminated foods, especially milk, fish and meat products. PCBs can cross the placenta causing prenatal exposure in the developing foetus and accumulates in breast milk. Literature suggests that compared to exposures later in life, foetuses and children are much more susceptible to the neurotoxic effects of PCB exposure, especially prenatally and possibly also in the first years of life [2]. The organochlorine pesticides HCB and DDE also are classified as persistent organic pollutants. and endocrine disruptors [3, 4]. Also animal data [5] have shown neurotoxic effects of PCB exposures. The neurotoxicity of PCBs may rest on many different mechanisms, including disruption of thyroid function, altering several neurotransmitter signalling pathways e.g. serotonergic, glutaminergic, GABA-ergic (dopaminergic and γ-aminobutyric acid) [4, 6–8]. Also, interference with intracellular Ca^2+^ dynamics may lead to altered dendritic arborisation and synaptogenesis [9].

Play behaviour (masculine/feminine) has been reported to change in children according to the level of prenatal PCB exposure, although in the opposite direction for boys and girls [10]. Gender specific effects might also occur with respect to cognition as higher exposed boys did worse on constructive tasks in the Yucheng study [11]. PCB’s, and other endocrine disrupting substances can disrupt brain sexual differentiation and may affect brain development in a gender specific way [12, 13].

In this study, part of the Flemish Environmental Health Survey (2002–2006), we measured internal exposure in cord blood to PCBs, compounds with ‘dioxin-like’ activity, chlorinated pesticides [DDE and hexachlorobenzene (HCB) and we also assessed IQ, behaviour, temperament and milestones in motor development up to 36 months of age. Much attention was paid to confounding factors including, amongst others, assessment of the environmental support, personality traits and intelligence of the mother.

We hypothesized that known effects on development and sex differences in effect were likely to occur and especially that gender specific play behavioural patterns would be influenced by prenatal exposure levels of PCB’s and CALUX-TEQ.

## Methods

### Study design and study population (participants)

The study was set up as an observational prospective cohort study. Internal exposure of neonates was measured at birth using cord blood. At age 12, 24 and 36 months emotional and behavioural problems were assessed, and at 36 months a neurobehavioral evaluation of mother and child was performed.

Participants were recruited as part of an Flemish Environmental Health Survey (2002–2006) in Flanders. Between September 2002 and February 2004, mother-child pairs from the general population were recruited through 26 maternities which were selected by stratified sampling in eight study areas. The study areas included two urban areas (Antwerp and Ghent), an area characterized by fruit orchards, a rural area and four types of industrial areas (harbour, non-ferrous smelters, chemical industry and household waste incinerators). The selected areas contained 20% of the inhabitants of Flanders. Pregnant mothers could be included if they lived for at least five years in the area of interest, if they gave informed consent, and if they were able to fill out Dutch questionnaires. Participants in the neurobehavioral part of the study were recruited in the rural areas and in the areas around the harbours, the waste incinerators, and the areas near the non-ferrous smelters.

The study group was selected based on following inclusion criteria by the co-workers of the Provincial Institute of Hygiene -Antwerp: pregnancy without major complications (eclampsia, risk of imminent preterm birth), born at term, no major congenital abnormalities or diseases, no twins, no abnormal or asymmetrical reflexes during standard neurological screening during the first days and the children having only Dutch as their mother-tongue to prevent differences in language development. Informed consent was asked to participants whom were eligible in the sampled areas. At the end, 214 baby-mother pairs had given informed consent. Numbers were diminished because biomonitoring results could not be obtained in all participants (between *N* = 148 CALUX-TEQ and *N* = 206 DDE, see Table 2). One hundred and twenty nine pairs in which biomonitoring results were available, could be fully followed up until the age of 36 months (60,3%). The number of participants in each analysis may be lower as not all tests could be completed in all children due to various factors (e.g. tiredness of the child, timing issues, cooperation of the child, failure of test systems).

### Blood sampling and measurements

#### PCBs, Calux-TEQ, DDE, HCB

Cord blood was aliquoted and plasma was separated by centrifugation within one day in either the maternity or blood bank laboratories. The aliquoted samples were kept in the refrigerator for maximal one week. Afterwards they were put at − 20 °C until analysis. In cord plasma, marker polychlorinated biphenyls (PCB 138, 153 and 180), PCB 118 and 170 which may have more neurotoxic potentials, chlorinated pesticides [DDE and hexachlorobenzene (HCB)], and dioxin-like compounds (CALUX® assay) were analysed. The PCBs and chlorinated pesticides were analysed by GC/ECD using the method of Gomara et al. [18]. The analyses were performed by two labs. Both laboratories participated in the AMAP proficiency testing scheme (Institute National de Santé Publique, Quebec, Canada). The measurement uncertainty (sum of systematic error and two times the reproducibility) was estimated from the results of the ClinChek and AMAP samples, and ranged between 21 and 34% for all the compounds except for HCB (64%). The limit of detection for all chlorinated compounds was 0.02 μg/L. Routinely measured cholesterol and triglycerides were used to express the results on a lipid weight basis [19]. Exposure to dioxin-like compounds was assessed via the CALUX® assay, based on in vitro activation of the aryl hydrocarbon receptor (AhR) of cultured H4IIE rat hepatoma cells by the dioxin-like compounds present in 5 mL cord plasma (BioDetection Systems BV, Amsterdam, The Netherlands). The extraction and clean-up procedures were performed as described by Koppen et al. [20]. The limit of detection was calculated as the light signal measured from the dimethyl sulfoxide-control plus 3 times its standard deviation on each well plate (= 16 pg CALUX-TEQ/g cord plasma fat).

Lead (Pb) and cadmium (Cd) were measured in cord blood using standard clinical biological techniques.

### General history, medical file data and general health questionnaires

#### Before birth

Shortly after birth/delivery, data regarding the child, data on life style of the parents and information about the current and former pregnancies were obtained through a general self-assessment questionnaire. These data and questionnaires were collected by trained staff members of the Provincial Institute for Hygiene and Epidemiology (V. Nelen). Women were asked whether they had had any infections, hypertension, diabetes or other diseases and/or complications during pregnancy (0/1), whether they had used medication (0/1), alcohol (0/1) or drugs (0/1), or had been smoking (0/1) during pregnancy. Almost all mothers reported to have taken some vitamin supplementation throughout their pregnancy.

#### At birth

Data regarding mother and child were also collected in the medical files of the hospital: data regarding the mother were medical history of the mother, weeks of pregnancy, complications during birth (0/1), age of the mother, congenital diseases (0/1) and breastfeeding (0/1). All children included were born at term (> 36 weeks). Data regarding the child were weight, length, Apgar scores, standard medical and neurological examination as performed by a specialized paediatrician. Two children had an asymmetrical Babinski sign, which were excluded. Four children had minor birth defects (a supplementary finger, a small skin-patch, minor dysplasia of the outer ears, one foot with moderate valgus malformation), which were considered as without significance. One child suffered from hydronephrosis and was excluded. All the other clinical signs were normal in all babies. After screening the files one baby was going to be raised in a foreign language and was excluded. In total, 202 mother-children pairs met all the inclusion criteria. If the Apgar score at the beginning (1 min) was 6 or less and/or other signs of foetal asphyxia were present (meconium stained amniotic fluid, aspiration, abnormal foetal heart rate, neonatal resuscitation) the baby was considered to have had perinatal asphyxia (0/1).

The first month (4–6 weeks after delivery) all mothers received a supplementary questionnaire. This questionnaire repeated and extended the questions used in the general questionnaire at birth as an extra control on the data. Semi-quantitative ethanol consumption (g ethanol/week) was calculated.

#### After birth

In the first year the mothers filled out a monthly written questionnaire (by post) on child development, feeding, diseases of the child, imported life events, medication, smoking, alcohol or drug use of the mother. After the first year this was repeated every three months (by post) until at the age of 36 months the child was investigated. Additionally, important life events and stress factors were yearly questioned with the STAI (State-Trait Anxiety Inventory).

### Neurobehavioral test methods and neurobehavioral questionnaires

**Fig. 1:**
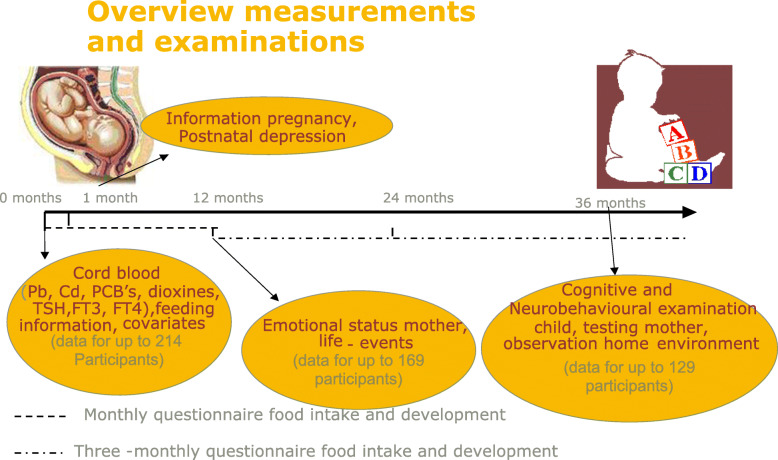
Overview and timeline of the study

An overview of the tests is shown in Tables 1, 2 and Fig. 1.

**Table 1:** Children: tests on behaviour, motor development, language skills, intelligence

Questionnaires	Parameter	Positive β indicates more or better	Age of child
Bayley mental scale [21] Bayley motor scale [21]	Visual and auditory discernment, eye-hand coordination, imitation, language development, memory and problem solving capacity. Mean score for the general population of 100 (± SD 15). Motor development. Mean score for the general population of 100 (± SD 15).	Cognitive development Motor development	36 months
Infant Behaviour Questionnaire (IBQ) [22]: Temperament as observed by the mother	Scale 1 = stress and latency to approach sudden or novel stimuli Scale 2 = smiling and laughter Scale 3 = distress to limitations Scale 4 = activity level Scale 5 = duration of orienting	Fear responses. Smiling or laughter from the child in general caretaking and play situations. Persistence and goal oriented behaviour. Gross motor activity: movement of arms and legs, squirming and locomotor activity. Habituation: more attention to and/or interaction with a single object for extended periods of time.	12 months
Milestones: developmental abilities	1 = grasping 2 = rolling over 3 = sitting up 4 = crawling 5 = first steps alone	Time needed to achieve the milestone: Grasping and picking up objects. Learning to flip over from his back to his tummy and vice versa. To sit up without support. Crawling. First steps alone unaided (first steps alone).	0–36 months
**Tests**	**Measurement type**	**Positive β indicates a higher capacity in terms of**	**Age of child**
Reynell Language Developmental Scale (RTOS): receptive language development level measured by oral instructions to carry out small tasks while playing with toys [23].	Verbal comprehension scale	Receptive language development.	36 months
Snijders-Oomen non-verbal intelligence test (SON 2.5–7), [24]: Intelligence: reasoning and visuospatial abilities	Three intelligence quotients (IQ) can be calculated with this intelligence tests with a mean score for the general population of 100 (± SD 15) Total intelligence scale: SON-IQ Performance Scale (PS) IQ Reasoning Scale (RS) IQ	(Nonverbal) intelligence (IQ). Performance IQ: better in making puzzles, mosaics and patterns. Reasoning IQ: better in categories, situations and analogies.	36 months
Observation of toy preference: 7 min. Lasting observation of masculine/feminine play behaviour [25, 26]	Masculine play behaviour Feminine play behaviour Non-gender specific play behaviour Switching attention	Time playing with masculine toys: little cars, fire truck, construction blocks, toy gun. Time playing with feminine toys: little dolls, hair doll, baby doll, kitchen accessories. Time playing with non-gender specific toys: puzzle, book The number of times the child switches between toys.	36 months
NES 3 (Neurobehavioral Evaluation System) [27]	Continuous Performance Test	Computerised vigilance task adapted according to Patandin et al., [28].	36 months

**Table 2:** Mothers: Tests concerning caring environment, intelligence, anxiety

Tests	Measurement type	Time of assessment
HOME [14, 15]	Home observation for measurement in the environment	36 months
WASI [16]	Wechsler Abbreviated Scale of Intelligence	36 months
STAI [17]	Trait and state anxiety measurement	12 months

At the time the child reached the age of 12, 24 and 36 months, the mother and pre-school playgroup teacher completed questionnaires regarding possible emotional and behavioural problems [Infant Behaviour Questionnaire (IBQ) [22]. The response rate of the IBQ at 24 months was too low to include the results of the analyses (< 40%).

At the time the child reached the age of 36 months, an appointment was made to carry out the neurobehavioral evaluation of mother and child and the HOME assessment (HOME, 1988).

All psychological tests and questionnaires used in this study are validated in Flanders for clinical use and are suitable for use in a research context. The NES (Neurobehavioral Evaluation System; [27]) was adapted according to the attentional task described by Patandin et al. [28] (catch the kitten). The tests were administered by trained test leaders with substantial experience in psychological testing of children and adults. The tests were divided over two test sessions and took place in a quiet room.

The Bayley Scales measure the mental and motor development and test behaviour of infants from one to forty-two months of age. The scales have been used extensively worldwide to assess the development of infants. The examiner presents a series of test materials to the child and observes the child’s responses and behaviours. The test contains items designed to identify young children at risk for developmental delay. In this study, mental en motor scales were assessed according to Van der Meulen et al. [21]. In the Mental scale, several types of abilities are evaluated: sensory/perceptual acuities, discriminations, and response; acquisition of object constancy; memory learning and problem solving; vocalization and beginning of verbal communication; basis of abstract thinking; habituation; mental mapping; complex language; and mathematical concept formation. In the Motor scale, the degree of body control, large muscle coordination, fine motor skills of the hands and fingers, dynamic movement, postural imitation, and the ability to recognize objects by sense of touch (stereo gnosis) were assessed.

Home Observation for Measurement of the Environment (HOME) is a descriptive profile which yields a systematic assessment of the caring environment in which the child is reared. The primary goal of the instrument is to measure, within a naturalistic context, the quality and quantity of stimulation and support available to a child in the home environment. Its focus is on the experience of the child in the home environment, the child as an active recipient of inputs from objects, events and transactions occurring in connection with the family surroundings [14, 15].

The Wechsler Abbreviated Scale of Intelligence (WASI, [16]) has been used in this study to obtain a reliable measure of intellectual ability of the mother. The WASI assessment provides traditional verbal, performance and full scale IQ scores.

A nominal STAI score was computed as a marker of prenatal stress [17]. The trait score of the STAI scale has proven to be stable across years, whereas the state score is more vulnerable to acute mood changes. Therefore a 0/1 variable was created, based on the P90 of STAI trait score (measured at 12 months).

### Statistical analysis

The relation between neurobehavioral development, including play behaviour, and the prenatal exposure levels of PCB and dioxin-like compounds, DDE, HCB and interaction effects between the prenatal exposure levels of sum PCB and DDE or HCB were analysed in the total group and in boys and girls separately.

Database management and statistical analyses were performed with the SPSS program.

With ANCOVA analysis the difference between the different test-leaders (scoring and testing) was checked, using the same covariates as for the stepwise regression analysis (see below). No significant differences were found (*p* > 0,050) (multiple linear regression).

Correlation analysis of the covariates in our study group, showed that the highest education level of the parents was very highly correlated with the total IQ of the mother (r^2^ = .52; *p* < .001) and parity was highly correlated with the HOME score (r^2^ = .34; *p* < .05). Highest education level and parity were therefore not included in the regression analyses.

Stepwise regression was performed in the total group using the cord blood concentrations of the sum of the marker polychlorinated biphenyls PCBs 138, 153 and 180 (sum PCB), dioxin like substances measured with the Calux test, DDE and HCB as biomarkers of exposure. As covariates for the neurobehavioral test data and questionnaire results, we used total IQ of the mother (WASI), gender, mother’s age at birth, smoking during pregnancy, alcohol use during pregnancy, neonatal asphyxia, medication use during pregnancy, infections or other interfering diseases during pregnancy, breastfeeding, STAI (trait, as a marker of prenatal stress), and total score of the HOME. Total IQ of the child (SON) was also taken into account for the stepwise multiple regression analyses of the questionnaire results. Stepwise regression analysis concerning gender-specific behaviour was done in the total group and in boys and girls separately. Gender specific results are only presented when statistically significant.

As group numbers differed over different tests, the number of subject is mentioned in every analysis.

In order to look at possible interaction and/or addition effects, the test and scale scores were transformed into standardised (z) scores. All potential factors were brought into the model (stepwise Multiple Linear Regression).

## Results

### Personal characteristics and measured parameters of participants

The personal characteristics and the data concerning measured parameters for all of the participants are summarized in Tables 3 and 4. No significant differences were noted in organochlorine cord blood concentrations between boys and girls (see supplementary material Table 1). No correlation was found between the organochlorines and the other parameters mentioned in Tables 3 and 4, except for a negative correlation of Pb with HCB (r2 = − 0,146, *p* = 0,045), and Pb with Calux-TEQ (r2 = − 0,191, p = 0,025).

**Table 3:** Characteristics and exposure of partcipants

Total group ***N*** = 214					
	N	Mean	Minimum	Maximum	Std. Deviation
HOME	127	47,7	28	55	4,3
Parity	206	1,6	1,0	4,0	0,8
Total IQ mother (WASI)	129	103,9	75	136	12,1
Highest education level of both parents	206	4,3	0	6	1,3
Age of mother at birth	206	29,5	20,3	41,6	3,9
BMI child at birth	206	13,6	9,8	20,6	1,4
SON, Total IQ-scale	125	113,7	70	150	13,6
Total STAI	169	0,1	0	1	0,4
Sum PCB ng/g total lipid *	198	87,9	2,8	305,6	59,6
PCB 118 ng /g total lipid	198	14,9	0	71,1	11,3
PCB 170 ng/g total lipid	197	8,9	0	55,6	7,4
Calux-TEQ pg/g total lipid	148	30,1	5,1	130,2	20,5
DDE ng/g total lipid	206	198,2	8,2	1274,5	193,5
HCB ng/g total lipid	197	27,8	2,4	132,1	20,4
Lead μg/L	201	19.2	1.0	87.3	15.8
Cadmium μg/L	201	0.7	0.1	13.9	1.1

HOME, WASI, SON total IQ-scale, Total STAI: see overview tests in Tables 1 and 2

Sum PCB, PCB 118, PCB 170, Calux-TEQ, DDE-conc., HCB-conc.: see “Blood sampling and measurements”

*Sum PCB = PCB 138+ PCB 153+ PCB 180

**Table 4:** Gender, events during pregnancy and at birth (N = 214)

	Number (%)	Missing data Number (%)
Boys	107 (50,0%)	0
Breastfeeding (0/1)	70 / 130 (33,0% / 61,3%)	12 (5,7%)
Smoked during pregnancy (0/1)	185/25 (87,3% / 11,8%)	2 (0,9%)
Alcohol use during pregnancy (0/1)	197 / 15 (92% / 7,1%)	2 (0,9%)
Asphyxia baby (0/1)	189 / 23 (89,2% / 10,8%)	0

### Description of neurobehavioral characteristics

Observed neurobehavioral characteristics are summarized in Table 5.

**Table 5:** Descriptive results: behaviour, motor development, language skills, intelligence

	N		Mean	Median	Std. Deviation	Minimum	Maximum
	Valid	Missing					
Bayley Scales of infant Development II NL: Mental Scale Index score	112	102	109,51	108,00	15,14	69	163
Bayley Scales of infant Development II NL: Motor Scale index score	93	121	101,97	101,00	12,78	79	137
Snijders-Oomen Non-Verbal Intelligence test: Performance Scale	125	89	108,66	110,00	15,92	67	148
Snijders-Oomen Non-Verbal Intelligence test: Reasoning Scale	125	89	115,13	115,00	12,50	66	150
Snijders-Oomen Non-Verbal Intelligence test: Total Scale	125	89	113,72	114,00	13,59	70	150
Reynell Language Development Scale: Language Development Total score (language comprehension)	101	113	102,80	104,00	17,82	59	142
milestones: smiling (months)	176	38	1,44	1,00	1,01	1	9
milestones: reach for and take a toy (months)	180	34	2,91	3,00	1,19	1	8
milestones: rolling over (months)	156	58	5,30	5,00	1,66	1	10
milestones: sit up straight (months)	154	60	7,29	7,00	1,44	4	15
milestones: crawling (months)	130	84	9,25	9,00	2,15	4	21
Milestones: first steps alone	69	145	13,06	14,00	2,57	8	20
IBQ 12 mths: Stress and Latency to Approach Sudden or Novel Stimuli	151	63	2,81	2,81	,81	1,20	4,58
IBQ 12 mths: Smiling and Laughter	151	63	5,41	5,47	,74	3,36	7,00
IBQ 12 mths: Distress to Limitations	151	63	3,04	3,00	,70,412	1,33	5,05
IBQ 12 mths: Activity Level	151	63	3,32	3,29	,88	1,24	5,47
IBQ 12 mths: Duration of Orienting	150	64	3,41	3,41	,947	1,36	6,29
Masculine play behaviour (%)	100	114	43,68	37,67	31,08	0,00	100,00
Feminine play behaviour (%)	100	114	41,51	36,99	30,39	0,00	100,00
Non-gender specific play behaviour (%)	100	114	14,81	9,61	18,31	0,00	94,00

There was no significant difference in any of the neurobehavioral outcome variables regarding the different regions (*p* > 0,050) (multiple linear regression).

### Associations with neurobehavioral characteristics using questionnaires

Associations with neurobehavioral characteristics measured using questionnaires are summarized in Table 6.

**Table 6:** Associations between exposure and behaviour assessed through IBQ (beta coefficients)

IBQ	12 months					
	N	scale 1 (stress)	scale 2 (smiling and laughter)	scale 3 (distress to limitations)	scale 4 (activity)	scale 5 (duration of orienting)
**Sum PCB** ng/g lipid	T = 96	−.081	−.164	.014	.047	−0.125
Girls	♀ = 44	NS	NS	NS	NS	NS
Boys	♂ = 52	NS	NS	NS	NS	NS
**Calux-TEQ** pg/g lipid	T = 68	−.247*	.117	.048	.119	.085
Girls	♀ = 37	NS	NS	NS	NS	0.468**
Boys	♂ = 31	NS	NS	NS	NS	NS
**PCB-118** ng/g lipid	T = 95	−.092	.007	−.201*	−.131	−.061
Girls	♀ = 51	NS	NS	NS	NS	NS
Boys	♂ = 44	NS	NS	--0.379*	NS	NS
**PCB-170** ng/g lipid	T = 95	.007	.187	−.103	−.054	−.083
Girls	♀ = 51	NS	NS	NS	NS	NS
Boys	♂ = 44	NS	NS	NS	NS	NS
**HCB** ng/g lipid	T = 93	−.150	.193*	−.022	.045	.182
Girls	♀ = 50	NS	NS	NS	NS	0.343*
Boys	♂ = 43	NS	0.386*	NS	NS	NS
**DDE** ng/g lipid	T = 100	−.201*	.071	−.147	.103	−.158
Girls	♀ = 54	−0.299*	NS	NS	NS	−0.308*
Boys	♂ = 46	NS	0.347*	NS	NS	NS
Sum PCB*HCB	T = 93	−.079	−.046	−.048	−.034	.031
Girls	♀ = 50	NS	NS	NS	NS	NS
Boys	♂ = 43	NS	NS	NS	NS	NS
Sum PCB*DDE	T = 96	.026	.078	.054	.159	.078
Girls	♀ = 52	NS	NS	NS	NS	NS
Boys	♂ = 44	NS	0.376*	NS	NS	NS

IBQ and IBQ scales: see overview tests in Table 1

PCBs, Calux-TEQ, DDE, HCB: see blood sampling and measurements

Su PCB*HCB, sum PCB*DDE: see statistical analysis

* = 0.050 ≤ *p* < 0.10, ** = 0.010 ≤ *p* < 0.050, *** = 0.001 ≤ *p* < 0.010

In boys and girls together higher CALUX-TEQ and DDE values were associated with a decrease in stress and latency to approach sudden or novel stimuli (scale1), HCB was associated with an increase in smiling and laughter (scale 2) and PCB-118 was associated with a decrease in distress to limitations (scale 3).

In girls, DDE was associated with a decrease in stress and latency to approach sudden or novel stimuli (scale 1) and a decrease duration of orienting (scale 5), and CALUX-TEQ and HCB were, contrary to DDE, associated with an increase in duration of orienting (scale 5).

In boys, higher internal exposure levels to HCB, DDE and the factor sum PCB*DDE were associated with an increase in smiling and laughter (scale 2), and PCB 118 was associated with an increase in distress to limitations. Interestingly, different significant associations between internal exposure to organochlorine compounds and temperament as observed by the mother were observed for boys and girls.

### Associations with differences in milestones

Associations with differences in milestones are summarized in Table 7.

**Table 7:** Associations between exposure and motor development in terms of milestones (beta coefficients)

Milestonesss					
Total Group	Grasping T = 109 ♀ = 56 ♂ = 53	rolling over T = 98 ♀ = 50 ♂ = 48	sitting up T = 99 ♀ = 50 ♂ = 49	Crawling T = 46 ♀ = 28 ♂ = 18	First steps alone T = 46 ♀ = 28 ♂ = 18
Sum PCB ng/g lipid	−.178	.020	.212	.230	.338**
Girls	NS	NS	NS	NS	NS
Boys	−.371*	−.427*	NS	NS	NS
Calux-TEQ pg/g lipid	.043	−.015	.015	.145	.078
Girls	NS	NS	NS	NS	NS
Boys	NS	NS	NS	NS	NS
PCB-118 ng/g lipid	.013	−.090	−.064	−.016	.451***
Girls	NS	NS	NS	NS	NS
Boys	NS	NS	NS	NS	.577**
PCB-170 ng/g lipid	−.081	−.043	.049	.193	.366**
Girls	NS	NS	NS	NS	NS
Boys	NS	NS	NS	NS	.378*
HCB ng/g lipid	.049	.039	.049	.175	.330**
Girls	NS	NS	NS	NS	NS
Boys	NS	NS	NS	NS	.450*
DDE ng/g lipid	.082	.019	−.260***	.028	.119
Girls	NS	NS	NS	NS	NS
Boys	NS	NS	−.296*	NS	.642**
Sum PCB*HCB	−.065	−.101	−.032	.278**	.463***
Girls	NS	NS	NS	.327*	NS
Boys	NS	NS	NS	NS	.538**
Sum PCB*DDE	−.183	−.077	−.163	.279*	.343**
Girls	−.312*	NS	NS	NS	NS
Boys	NS	NS	NS	NS	.630**

Milestones: see overview tests in Table 1

PCBs, Calux-TEQ, DDE, HCB: see blood sampling and measurements

Sum PCB*HCB, sum PCB*DDE: see statistical analysis

* = 0.050 ≤ p < 0.10, ** = 0.010 ≤ p < 0.050, *** = 0.001 ≤ p < 0.010

In the first 36 months (girls and boys together), increasing levels of sum PCB as well as increasing levels of individual PCBs (PCB118, PCB 170) and of HCB were associated with a significant later onset of first steps alone. The interaction between PCBs and pesticides was not only associated with a delay in the first steps, but was also associated with a delay in the age of crawling. In boys only, earlier grasping and earlier rolling over tended to be associated with sum PCB (Table 7). In girls sum PCB*DDE tended to be associated with earlier grasping. CALUX-TEQ was not associated with a negative effect on milestones in the total group. Higher DDE concentrations were significantly associated with delayed first steps alone in boys. Both in boys and in the total group a significant association was found between higher prenatal DDE concentrations and ability to sit up earlier.

Effects seem to be more pronounced in boys than in girls, except that in girls delayed crawling showed a positive association with the interaction of sum PCB*HCB and that the ability to grasp showed a positive association with higher sum PCB*DDE.

### Cognitive tests and behavioural observations

Associations with differences in cognitive tests are summarized in Table 8.

**Table 8:** Associations between exposure and cognitive functioning (beta coefficients)

Cognitive tests					
	**N**	**RTOS** (language development) T = 97 ♀ = 50 ♂ = 47	**SON-RS IQ** (Reasoning) T = 125 ♀ = 62 ♂ = 63	**SON-PS IQ** (performance) T = 125 ♀ = 62 ♂ = 63	**SON-TIQ** (total intelligence) T = 125 ♀ = 62 ♂ = 63
Sum PCB ng/g lipid	99	−.103**	−.036	−.054	−.043
Girls		NS	NS	NS	NS
Boys		NS	NS	NS	NS
Calux-TEQ pg/g lipid	60	−.267**	−.002	−.042	−.010
Girls		NS	NS	NS	NS
Boys		NS	NS	NS	NS
PCB-118 ng/g lipid	99	−.125*	NS	NS	NS
Girls		NS	NS	NS	NS
Boys		NS	−.353**	−.401***	−.424***
PCB-170 ng/g lipid	98	−.243**	−.050	−.107	−.083
Girls		NS	NS	NS	NS
Boys		NS	NS	NS	NS
HCB ng/g lipid	98	−.262**	−.212	−.055	−.147
Girls		NS	NS	NS	NS
Boys		NS	NS	NS	NS
DDE ng/g lipid	79	−.028	.052	−.009	.026
Girls		NS	NS	NS	NS
Boys		NS	NS	NS	NS
Sum PCB*HCB	97	−.327***	−.168	−.072	−.121
Girls		NS	NS	NS	NS
Boys		NS	NS	NS	NS
Sum PCB*DDE	79	−.245**	−.048	.045	−.010
Girls		NS	NS	NS	NS
Boys		NS	NS	NS	NS

RTOS, SON-RS IQ, SON-PS IQ, SON-TIQ: see overview tests in Table 1

PCBs, Calux-TEQ, DDE, HCB: see blood sampling and measurements

Sum PCB*HCB, sum PCB*DDE: see statistical analysis

* = 0.050 ≤ p < 0.10, ** = 0.010 ≤ p < 0.050, *** = 0.001 ≤ p < 0.010

At 36 months, increasing levels of sum PCB as well as increasing levels of individual PCBs (PCB118, PCB 170) and CALUX-TEQ were associated with significant lower performance on language development (RTOS). Compared to the lowest sum PCB exposed children, the highest exposed children had RTOS scores that were 38% lower. Increasing concentrations of HCB and higher values for the interaction between PCB and pesticides were associated with the same effect. One of the neurotoxic compounds, PCB118, was associated with a significant deterioration, in boys only, of reasoning (SON-RS IQ), performance (SON-PS IQ) and total IQ (SON-TIQ). The interaction factor sum PCB*HCB may have an additive effect. DDE does not seem to be associated with an adverse effect, except in interaction with sum PCB (Table 8), possibly suggesting a synergistic effect.

The NES results were not analysed (see discussion).

### Associations with observed differences in play behaviour

Associations with observed differences in behaviour are summarized in Table 9.

**Table 9:** Associations between exposure and play behaviour (beta coefficients)

		Girls				Boys			
	**girls/boys** **N**	**masculine** **play behaviour**	**feminine play behavior**	**non-gender specific play behaviour**	**switching attention**	**masculine** **play behaviour**	**feminine play behaviour**	**non-gender specific play behaviour**	**switching attention**
**Sum PCB ng/g lipid**	47/37	−.247*	−.121	.250*	−.342**	−.269*	−.002	.266*	−.383**
**Calux-TEQ pg/g lipid**	47/37	−.010	−.162	.313*	.126	−.199	.257	.014	−.244
**PCB-118 ng/g lipid**	45/39	−.313**	.202	.052	.273	−.326**	−.087	.361**	−.325**
**PCB-170 ng/g lipid**	47/37	−.031	−.181	.314**	−.035	−.286*	−.096	.278*	.091
**HCB ng/g lipid**	51/43	−.066	.114	−.100	−.091	−.374**	−.027	.363**	−.163
**DDE ng/g lipid**	53/43	.307	−.248	−.047	−.041	−.331**	.228	.145	−.006
Sum PCB*HCB	47/37	.066	−.101	.075	.081	−.079	.056	.051	−.106
Sum PCB*DDE	47/37	.103	−.121	.054	−.031	.146	−.034	−.166	.161

Play behaviour: see overview tests in Table 1

PCBs, Calux-TEQ, DDE, HCB: see blood sampling and measurements

Sum PCB*HCB, sum PCB*DDE: see statistical analysis

* = 0.050 ≤ p < 0.10, ** = 0.010 ≤ p < 0.050, *** = 0.001 ≤ p < 0.010

In the toy preference task at 36 months, several significant associations were observed for as well girls as boys. Higher sum PCB exposed children alternate less between toys. They play more with a puzzle or book (non-gender specific play behaviour) compared to children with lower prenatal sum PCB-levels and they display less masculine play behaviour. An increase in concentration of sum PCB of 58,82 ng/g lipids is associated with one time less switching between toys during 7 min. Considering the observed range of sum PCB (2,81 till 305,56 ng/g lipids) the highest exposed child switches toys almost once per minute less compared to the lowest exposed child. Switching in this case means also crawling, first steps alone and turning, as toys were displayed in a semi-circle. PCB 118 exposure was associated with a decrease in masculine play behaviour in as well girls as boys. PCB 17 exposure was associated with an increase in non-gender specific play behaviour in as well girls as boys.

As to significant association observed only in girls, CALUX-TEQ exposure was associated with an increase in non-gender specific play behaviour.

As to significant associations observed only in boys, PCB118 exposure was associated with an increase in non-gender specific play behaviour, and a decrease in switching between toys. PCB 170 exposure was associated with a decrease in masculine play behaviour. HCB exposure was associated with a decrease in masculine play behaviour and with an increase in non-gender specific play behaviour. DDE exposure was associated with a decrease in masculine play behaviour.

As to the effect of siblings on the play behaviour of the tested children, we noted that 13 of the boys had a sister, and 12 of the girls had a brother. As can be seen from supplementary material Table 3, having a sister did not weaken the associations between internal exposure and play behaviour in boys. Neither did having a brother weaken the associations between internal exposure and play behaviour in girls as shown in supplementary material Table 4.

As can be seen from Table 9, observed associations were more pronounced in boys than in girls. In boys, sum PCB exposure was associated with a more pronounced diminishment in switching behaviour compared to boys and girls considered together. An increase of 37,04 ng/g lipids in the sum PCB levels is associated with 1 switch between toys less per 7 min, 64% less playing time with boys-specific toys and 62% more with non-gender specific toys.

## Discussion

Limitations of this study include the fact that the neurological examination at the time of birth, performed by a specialized pediatrician, was not performed by certified researchers. Also, although the children were examined at 36 months by specialized clinical psychologists, no complete medical assessment nor school results were available at the end of the study. As the Flemish Agency for Mental Health involved in the Study closed down many years ago and that many documents and computer files got lost, some details of the statistical models used are not available any more.

In this older cohort (2002–2004), representative for the Dutch speaking Flemish population, attention was paid to controlling for confounding factors, although not all confounding factors could be taken into consideration (e.g. the possible influence of air pollution on brain function) [29]. Analyses did not indicate bias by co-exposure of Pb and Cd, which were measured as ubiquitous neurotoxic environmental polluting metals in cord blood .

Comparing the exposure levels in our study with other studies is not straightforward because different parameters are measured in the different studies, and because different ratios were observed between maternal serum concentrations and cord serum concentrations. We made a tentative comparison summarized in supplementary material Table 2, which indicates that the internal exposure to PCBs in our study is situated in a middle range of internal exposures measured in other studies on general populations [28, 30–42], and is more than 20 times lower than in the Yucheng incident in Taiwan [42].

The results of all neurobehavioral tests and questionnaires are described extensively and are consistent with neurobehavioral effects reported before (2, review). Compared to the lowest exposed children, the highest exposed children had language comprehension scores (RTOS) that were 38% lower. One of the neurotoxic compounds, PCB118, was associated with a significant deterioration, in boys only, of reasoning (SON-RS IQ), performance (SON-PS IQ) and total IQ (SON-TIQ.

Our results resemble those reported by Rogan et al. [34], Boucher et al. [43], Berghuis et al. [31] in which it was shown that first milestones may be reached earlier (grasping, rolling over) but later milestones (first steps alone) may be delayed with higher prenatal exposures to PCBs and dioxin-like compounds. These both effects seem to be more pronounced in boys compared to girls (sum PCB, sum PCB*HCB, sum PCB*DDE, DDE, PCB118, PCB170).

The milestones like crawling and first steps alone are markers of motor development requiring balance and coordination, but also requiring active behaviour. Active motor behaviour is one of the six elements of temperament [44]. We measured temperament at 12 months of age as a basic characteristic of behaviour by the IBQ. Temperament is described to be influenced by heredity, maturation and experience [45, 46] but may also be influenced by prenatal toxic factors e.g. parental smoking [47]. Effects on temperament related to PCBs or dioxin-like compounds have not been frequently reported in toddlers [2]. Our results reported in Table 6. suggest that prenatal exposure to these endocrine disruptors might result in less distressed children. This could result in gradually less reactive children to stimuli and possibly underarousal patterns in higher exposed children [45]. Our results seem to be similar to those of Jacobson et al. [48], showing a rather reduced activity in 4-year old children associated with higher PCB levels.

We observed a less masculine, more non-gender specific, less active play behaviour with less switching between toys in association with PCB exposure, which was more pronounced in boys. An underarousal pattern may explain this observed reduced play activity as well as the observations made in terms of the milestones and IBQ.

Vreugdenhil et al. [10] also found a less masculinised play behaviour, although it was not statistically significant, and although in girls postnatal PCB exposure through breastmilk was positively related to more masculine play behaviour. Vreugdenhil et al. [10] used the PSAI questionnaire to measure sex linked play behaviour, which has some drawbacks [49]. In contrast to the PSAI, our experience with the Observation of Toy Preference is that this test is an easy to applicate, easy to interpret, parent independent measurement of gender-specific behaviour, with a high (0.99) inter-observer reliability.

Patandin et al. [38] described a play behavioural test with high and low level play episodes. High level play, number of episodes of non-play behaviour and period of time at non-play behaviour were significantly diminished in association with prenatal exposure to PCBs. Although the measurements are not totally comparable, they both measure adequate play behaviour which seems to be diminished in our and Patandin’s group. If this is a result of attentional deficit or due to reduced activity is a matter of debate [28, 48]. Patandin [28] did not found a relation between cord blood PCB concentrations and most attentional parameters (mean reaction time, slope of reaction time, Groninger Behaviour Observation Scale parents questionnaire) in 42 months old children, but measuring attention may not be valid under 4.5 years [50]. We tried to measure attention in 36 month old children by a test described by Jacobson et al. [51] and used by Patandin [28] and Stewart [52]. The frame of the continuous performance test of the NES3 was used to develop a test which might be feasible for 3 year old children. We noticed that most children didn’t complete the test (not fully validated in children of this age), probably because sustained attention cannot be reliably measured at this age (results not reported). However, diminished attention related to perinatal PCB exposures are frequently reported in older children (> 4 years) [2].

In contrast to others, we didn’t find any influence of breastfeeding [53–55].

Sex-linked differences as reported here, in other human studies [10, 11, 56–58] and in animal studies [59–62] might result from oestrogenic and anti-oestrogenic actions [4, 63, 64] as oestrogens are essential in the neurogenesis and functionality of neurons which are important in learning processes and behaviour such as anxious and gender-specific behaviour [5, 65]. In mammalians, the ontogenetic evolution towards the female phenotype appears in some way to be the default process and occurs to a large extent without sex hormone influence [12], while even small differences in levels and timing of exposure to hormones or endocrine disruptors may disrupt brain sexual differentiation [12]. The group of Andrea Gore showed in rats that PCBs cause changes in sexually-dimorphic social interactions and communications and that these changes were associated with (and probably rested on) modifications of gene expression in the medial preoptic nucleus, part of the social decision-making network in the brain. In females PCBs changed expression of several steroid hormone receptor and neuropeptide genes, whereas in males only one gene (the circadian gene *Per2*) was affected, although more behavioral changes were observed in males [66]. Even adult males and females do not necessarily react in the same way to exogenous substances. In the Flemish biomonitoring, changes in gene expression associated with internal exposure to DDE, hexachlorobenzene, marker PCBs and dioxin-like activity were predominantly in opposite direction for men and women [67].

## Conclusion

Our data confirm the observations that neurobehavioral development of young children is adversely influenced by environmental concentrations of organochlorine pollutants, especially in boys. Motor development, playing activity and cognitive development tend to be delayed or diminished. We found observation of play behaviour to be a reliable, easy to perform and sensitive test to detect neurotoxic effects of chemicals like PCB’s and dioxin-like compounds in very young children. On the basis of our results, we hypothesize that an underarousal pattern may play a role in the spectrum of effects measured in toddlers prenatally exposed to PCBs and dioxin-like compounds.

## Supplementary Information

**Additional file 1.**

**Additional file 2.**

**Additional file 3.**

**Additional file 4.**

## Data Availability

The aggregated data are publically available via the IPCHEM data platform (https://ipchem.jrc.ec.europa.eu/RDSIdiscovery/ipchem/index.html#discovery). The individual records can be requested via the procedures that are available on this portal (https://ipchem.jrc.ec.europa.eu/RDSIdiscovery/ipchem/index.html#showmetadata/FLEHS1REFNB).

## Abbreviations

BMI: Body Mass Index
CALUX-TEQ: Dioxin-like activity measured via the CALUX® assay
DDE: P,p’-dichlorodiphenyldichloroethylene
HCB: Hexachlorobenzene
HOME: Home Observation Measurement of the Environment
IBQ: Infant Behavior Questionnaire
IQ: Intelligence quotient
NES: Neurobehavioral Evaluation System
PCB: Polychlorinated biphenyls
POPs: Persistent organic pollutants
PS IQ: Performance Scale IQ (SON)
RS IQ: Reasoning Scale IQ (SON)
RTOS: Reynell TaalOntwikkelingsSchalen (language development)
SD: Standard Deviation
SON: Snijders-Oomen Niet-verbale intelligentietest (non-verbal intelligence)
SON-IQ: Total intelligence scale (SON)
STAI: State-Trait Anxiety Inventory
SumPCB: Sum of polychlorinated biphenyls
WASI: Wechsler Abbreviated Scale of Intelligence
