# Supplementary material for: Neurobehavioural and cognitive effects of prenatal exposure to organochlorine compounds in three year old children
Source: BMC Pediatr. 2021 Feb 26;21:99. doi: 10.1186/s12887-021-02533-2 (PMC7908674; doi:10.1186/s12887-021-02533-2)
Supplement: Supplementary file 1 — Additional file 1. [file 12887_2021_2533_MOESM1_ESM.docx]

**Supplementary material Table 1.**

**Organochlorine serum concentrations in cord blood from boys and girls**

|  | Boys | | | Girls | | |  |
| --- | --- | --- | --- | --- | --- | --- | --- |
|  | N | Mean ng/g lipids | Standard deviation | N | Mean ng/g lipids | Standard deviation | p |
| SumPCB/total lipid conc* | 100 | 91,2837 | 59,2978 | 98 | 84,5123 | 59,9485 | 0.43 |
| PCB 118/total lipid conc ° | 100 | 15,5358 | 10,8560 | 98 | 14,1647 | 11,7382 | 0.39 |
| PCB 170/total lipid conc | 100 | 9,0327 | 6,8510 | 97 | 8,8273 | 7,9902 | 0.85 |
| CaluxTEQ/total lipid conc | 74 | 31,1570 | 23,8221 | 74 | 29,0414 | 16,6247 | 0.53 |
| DDE-conc/total lipid conc | 104 | 213,8977 | 208,4954 | 102 | 182,1455 | 176,4493 | 0.24 |
| HCB-conc/total lipid conc | 100 | 29,0666 | 22,2490 | 97 | 26,4758 | 18,3555 | 0.37 |
