# Supplementary material for: Neurobehavioural and cognitive effects of prenatal exposure to organochlorine compounds in three year old children
Source: BMC Pediatr. 2021 Feb 26;21:99. doi: 10.1186/s12887-021-02533-2 (PMC7908674; doi:10.1186/s12887-021-02533-2)
Supplement: Supplementary file 2 — Additional file 2. [file 12887_2021_2533_MOESM2_ESM.docx]

**Supplementary material Table 2. Tentative comparison between exposure levels in some studies concerning PCBs and neuropsychic parameters**

| Study | Country | Mean/med | Parameter measured | Concentration measured in maternal serum ng/L | Concentration measured in cord blood ng/L | Concentration measured in maternal serum ng/g lipid | Concentration measured in cord blood ng/g lipid | Estimated cord blood level^a^ ng/g lipid (low estimate; high estimate) |
| --- | --- | --- | --- | --- | --- | --- | --- | --- |
| Zhang et al. (2017) | Ohio USA | median | SumPCB |  |  | 31.30 |  | 25.9;40.1 |
| Berghuis et al. (2018) | Netherlands | median | SumPCB |  |  | 319.0 |  | 263.5;408.3 |
| Kim et al. (2018) | Korea | median | SumPCB |  |  | 27.3 |  | 22.6;34.9 |
| Rosenquist et al. (2017) | Ukraine | median | PCB 153 |  |  | 27 |  | 53.1;82.3 |
| Rosenquist et al. (2017) | Greenland | median | PCB 153 |  |  | 107 |  | 210.4;326.1 |
| Kyriklaki et al. (2016) | Creta | median | SumPCB | 320.8 |  |  |  | 31.5;48.9 |
| Rogan et al. (1986) | North Carolina USA | median | PCB153 |  |  | 80 |  | 157.3; 243.8 |
| Jacobson & Jacobson (1996) | Michigan USA | median | PCB153 |  |  | 120 |  | 236.0;365.7 |
| Winneke et al. (1998) | Dusseldorf Germany | mean | SumPCB |  |  |  | 218.0 |  |
| Patandin et al. (1999) | Netherlands | median | SumPCB | 2,040 | 380 |  |  | 200.6;310.9 |
| Huisman et al. (1995a) | Netherlands | median | SumPCB | 2,040 | 380 |  |  | 200.6;310.9 |
| Huisman et al. (1995b) | Netherlands | median | SumPCB | 2200 | 430 |  |  | 216.3;335.2 |
| Steuerwald et al.(2000) | Faroe islands | Geometric mean | SumPCB | 1,120 |  |  |  | 110.1;170.7 |
| Guo et al., (2004) | Taiwan Yucheng incident | median | SumPCB | 26800 |  |  |  | 2,635.4;4,083.9 |
| This study | Flanders | median | SumPCB |  |  |  | 74,88 |  |

^a^ Estimated cord serum levels were calculated using the following assumptions: PCB 153 was considered to represent 42% of sumPCBs, based on observations in Belgium (see below); maternal serum was considered to contain 8.4g lipid per liter; for the ratio between concentrations in ng/glipid in cord blood versus ng/g lipid in maternal blood two values were used (0.826 and 1.28) as different values can be found in the literature (see below).

In Belgium PCB 153 accounted for 42.5% of all measured PCBs In femaleadolescents and for for 41.6 % of all measured PCBs in female adults (De Bont et al., 2004).

The ratio between sumPCB values in ng/g lipid in cord serum compared to maternal serum was about 1 in the study of Steuerwald et al. (2000), amounted to 0.82.6 in the study of Muckle et al., (2001) and to 1.28 in the study of Soechitram et al. (2004).
