# Supplementary material for: Neurobehavioural and cognitive effects of prenatal exposure to organochlorine compounds in three year old children
Source: BMC Pediatr. 2021 Feb 26;21:99. doi: 10.1186/s12887-021-02533-2 (PMC7908674; doi:10.1186/s12887-021-02533-2)
Supplement: Supplementary file 3 — Additional file 3. [file 12887_2021_2533_MOESM3_ESM.docx]

**Supplementary material Table 3. Boys. Effect of having a sister on some associations between internal exposure and play behaviour (Beta coefficients and p value)**

| Independent variables | **masculine**  **play behaviour** | **Feminine play behaviour** | **Ln non-gender specific play behaviour** | **switching attention** |
| --- | --- | --- | --- | --- |
| **Ln SumPCB ng/g lipid** | -16,07 (p=0.011) | 1,892 (p=0.70) | 0,7439  (p=0.018) | -2,670  (p=0,0072) |
| **Ln SumPCB ng/g lipid**  **Having a sister** | -15,94 (p=0.012) | 1,850  (p=0.70) | 0,7378  (p=0.019) | -2,666 (p=0.0071) |
| **LnPCB-118 ng/g lipid** | -13,68 (p=0.038) | -1,315  (p=0.80) | 0,840918  (p=0.0031) | -2,053 (p=0,063) |
| **LnPCB-118 ng/g lipid**  **Having a sister** | -14,2022 (p=0.032) | -3,154 (p=0.55) | 0,8605  (p=0.0024) | -2,032 (p=0.073) |
| **PCB-170 ng/g lipid** | -0,690 (p=0.33) | 0,1575  (p=0.75) | 0,0405  (p=0.29) | -0,236 (p=0.021) |
| **PCB-170 ng/g lipid**  **Having a sister** | -0,685 (p=0.33) | 0,1356  (p=0.78) | 0,0391  (p=0,32) | -0,235 (p=0.021) |
| **Ln HCB ng/g lipid** | -10,84 (p=0.052) | 1,584(p=0.69) | 0,3467  (p=0.12) | -1,334 (p=0.11) |
| **Ln HCB ng/g lipid**  **Having a sister** | -11,18 (p=0.045) | 0,7925 (p=0.84) | 0,3535  (p=0.17) | -1,316 (p=0.12) |
| **LnDDE ng/g lipid** | -10,63 (p=0.053) | 1,777 (p=0.68) | 0,4349  (p=0,010) | -2,299 (p=0.0064) |
| **ln DDE ng/g lipid**  **Having a sister** | -10,58 (p=0.055) | 1,230 (p=0.77) | 0,4347  (p=0.10) | -2,301 (p=0.0066) |
| **Calux-TEQ pg/g lipid** | -0,2988  (p=0.17) | 0,2641 (p=0.15) | 0,00228(p=0.83) | 0,0138 (p=0.69) |
| **Calux-TEQ pg/g lipid**  **Having a sister** | -0,2591 (p=0.24) | 0,1985  (p=0.26) | 0,00157  (p=0.88) | 0,0133(p=0.71) |
