# Supplementary material for: Neurobehavioural and cognitive effects of prenatal exposure to organochlorine compounds in three year old children
Source: BMC Pediatr. 2021 Feb 26;21:99. doi: 10.1186/s12887-021-02533-2 (PMC7908674; doi:10.1186/s12887-021-02533-2)
Supplement: Supplementary file 4 — Additional file 4. [file 12887_2021_2533_MOESM4_ESM.docx]

**Supplementary material Table 4. Girls. Effect of having a brother on some associations between internal exposure and play behavior (Beta coefficients and p value)**

| Independent variables | **masculine**  **play behavior** | **feminine play behavior** | **Ln non-gender specific play behaviour** | **switching attention** |
| --- | --- | --- | --- | --- |
| **Ln SumPCB ng/g lipid** | -10,88 (p=0,016) | 5,366 (p=0.31) | 0,7090  (p=  0,00043) | -1,709 (p=0,018) |
| **Ln SumPCB ng/g lipid**  **Having a brother** | -10,86 (p=0,015) | 5,349 (p=0.31) | 0,7368 (p=0,00026) | -1,707 (p=0.018) |
| **LnPCB-118 ng/g lipid** | -6,275 (p=0.10) | 7,653 (p=0.093) | 0,0595  (p=0.74) | -1,020 (P=0.27) |
| **LnPCB-118 ng/g lipid**  **Having a brother** | -6,432 (p=0.097) | 7,871 (p=0.085) | 0,0771  (p=0.68) | -1,061 (p=0.25) |
| **LnPCB-170 ng/g lipid** | -10,66 (p=0,046) | 4,549 (p=0.46) | 0,6706 (p=  0,00023) | -1,222 (p=0.23) |
| **LnPCB-170 ng/g lipid**  **Having a brother** | -11,40 (p=0,028) | 5,175 (p=0.39) | 0,6708  (p=  0,00040) | -1,227 (p=0.23) |
| **Ln HCB ng/g lipid** | -4,881 (p=0.20) | 5,391 (p=0.20) | 0,1525 (p=0,41) | -0,865 (p=0.31) |
| **Ln HCB ng/g lipid**  **Having a brother** | -5,503 (p=0.14) | 5,936 (p=0,16) | 0,1675 (p=0,38) | -0,909 (p=0.29) |
| **DDE ng/g lipid** | 0,0177 (p=0,31) | -0,0158 (p=0,42) | 0,00178 (p=0,091) | -0,00394 (p=0.37) |
| **DDE ng/g lipid**  **Having a brother** | 0,0178 (p=0.30) | -0,0159 (p=0.42) | 0,00185 (p=0,079) | -0,00395 (p=0.37) |
| **Calux-TEQ pg/g lipid** | -0,1053 (p=0,72) | -0,2057  (p=0.53) | 0,0222 (p=0,071) | 0,03157 (p=0.65) |
| **Calux-TEQ pg/g lipid**  **Having a brother** | -0,2095 (p=0.49) | -0,1781  (p=0.61) | 0,0246 (p=0,052) | 0,03742 (p=0.61) |
